# Psychophysiology of False Memories in a Deese-Roediger-McDermott Paradigm with Visual Scenes

**DOI:** 10.1371/journal.pone.0030416

**Published:** 2012-01-17

**Authors:** Ali Baioui, Wolfgang Ambach, Bertram Walter, Dieter Vaitl

**Affiliations:** 1 Bender Institute of Neuroimaging, University of Giessen, Giessen, Germany; 2 Institute for Frontier Areas of Psychology and Mental Health, Freiburg, Germany; Baycrest Hospital, Canada

## Abstract

Remembering something that has not in fact been experienced is commonly referred to as false memory. The Deese-Roediger-McDermott (DRM) paradigm is a well-elaborated approach to this phenomenon. This study attempts to investigate the peripheral physiology of false memories induced in a visual DRM paradigm. The main research question is whether false recognition is different from true recognition in terms of accompanying physiological responses.

Sixty subjects participated in the experiment, which included a study phase with visual scenes each showing a group of interrelated items in social contexts. Subjects were divided into an experimental group undergoing a classical DRM design and a control group without DRM manipulation. The control group was implemented in order to statistically control for possible biases produced by memorability differences between stimulus types. After a short retention interval, a pictorial recognition phase was conducted in the manner of a Concealed Information Test. Simultaneous recordings of electrodermal activity, respiration line length, phasic heart rate, and finger pulse waveform length were used. Results yielded a significant Group by Item Type interaction, showing that true recognition is accompanied by greater electrodermal activity than false recognition.

Results are discussed in the light of Sokolov's Orienting Reflex, the Preliminary Process Theory and the Concealed Information Test. Implications and restrictions of the introduced design features are critically discussed. This study demonstrates the applicability of measures of peripheral physiology to the field of false memory research.

## Introduction

Schematic knowledge assists in the integration of new and old information, yet it also leads to a vulnerability for false memories (i.e., mistaking new information as previously known). The authenticity of statements has been the focus of research on the detection of concealed information. Peripheral psychophysiological measures have been used to investigate whether statements are truthful or deceptive.

The main idea of this study was to differentiate true from false memories, similar to the differentiation of true and false statements from a psychophysiological detection-of-information perspective (see [1]). Hence, the main starting-point for differentiating true from false memories is the absence (false memory) or presence (true memory) of objective knowledge about an object.

### False memories in the DRM paradigm

In the *Deese-Roediger-McDermott (DRM) paradigm* [2], subjects learn lists of closely related words (*studied items*; e.g. “bed”, “pillow”, “sheet”) in a study phase. Later, in a recognition phase, subjects often falsely recognize words (*related lures*; e.g. “sleep”), which were not part of the previously learned lists but are strongly associated with these words. The external validity of the DRM paradigm was underlined by Clancy et al. [3]. They showed that susceptibility to false memories as evoked in DRM studies might be a feasible marker for proneness to false memories. In their study, people who reported to have memories of extremely improbable events (abduction by space aliens) showed higher false memory rates in a DRM experiment than controls. For an extensive review of the DRM paradigm and its applications, see [4].

While the bulk of DRM studies was conducted with words as stimuli, some researchers used serially presented pictures [5]–[8]. In these studies, the use of pictures led to relatively low false memory rates. Instead of a serial presentation of pictorial stimuli, Miller and Gazzaniga [9] showed all items of a category simultaneously by using visual scenes in one of their experimental conditions (picture condition). This led to robust false memory rates. Miller and Gazzaniga's [9] design has been successfully implemented in two clinical studies [10], [11]. In the healthy control groups of these studies (investigating posttraumatic stress disorder and schizophrenia patients as clinical groups), Miller and Gazzaniga's findings could be replicated. With a scenic presentation of stimuli some methodological problems of the original DRM design could be bypassed (e.g. primacy and recency effects in the study phase; see [2]: Experiment 1). Furthermore, pictorial stimuli might offer a higher ecological validity and a broader base for generalizations.

An inherent problem of the classical DRM design is the pre-selection of items serving as related lures. Stimuli are not randomly assigned to serve as related lures or studied items, respectively. This can lead to a priori differences in memorability of related lures and studied items [12]. Nessler et al. [13] argued that this could hamper studies investigating the psychophysiological indicants of false memories (e.g. event-related potentials). As a consequence, they suggested the selection of lures from a categorically associated list of items (“categorical design”; e.g. [8], [14], [13]). Disadvantage of this design are smaller false recognition rates [13]. Thus, in DRM studies with visual stimuli, in which false recognition rates are lower than in studies with textual stimuli, the implementation of a categorical design seems impracticable. Obviously, the applicability of a categorical design strongly depends on the nature of the stimuli.

We argue that this problem of the classical DRM design could also be approached by including a control group. In the control group, the subjects would actually view the related lures that are not shown in the experimental group. This permits the computation of between-group interaction effects that address such a memorability bias statistically. To our knowledge, this has not been implemented before in a DRM study.

### Psychophysiological differentiation of true and false memories

Several DRM studies examined the neurophysiological correlates of false memories and outlined the differential reactions accompanying true and false recognition using electroencephalography (e.g. [15]–[17], [13]), functional magnetic resonance imaging (e.g. [18]–[23]), and positron emission tomography [24]. For a review, see [25].

To our knowledge, measures of *peripheral* physiology have not yet been applied in a *DRM* study. Such an endeavor could however be promising: especially EDA is well suited to reflect whether a stimulus is known or unknown to a subject; this has been shown for pictorial as well as for verbal material (e.g. [26]) and also for semantically associated stimuli [27].

### Peripheral psychophysiology of object recognition

In general, autonomic measures reflect basal processes such as the orienting reflex [28], which is modulated by stimulus *intensity*, *significance*, and *novelty* [29]. The orienting reflex illustrates the relationship between information processing and peripheral psychophysiology. Habituation processes lead to smaller response amplitudes in peripheral physiological measures if a stimulus is presented repeatedly [29]. Any change of this stimulus (in terms of “novelty” or “intensity”) evokes an orienting response associated with an increased response amplitude [29]. Likewise, any stimulus with a *signal value* evokes an enhanced orienting response (significance; [30]–[33]). Sokolov [33] described that significance can be induced through classical conditioning. Maltzman [32] explained such an acquisition of stimulus significance by means of *cortical sets*. Cortical sets are “additional focused cortical activity elicited via conditioning, instructions, or prior experience” [34]. An activated cortical set can be interpreted as an unconditioned stimulus, the perceived stimulus as neutral/conditioned stimulus, and significance as conditioned response [32].

Bernstein et al. [35] conducted extensive research on the differential reactions of the different markers of peripheral physiology elicited by significance. They showed that time courses of electrodermal, cardiac, and eyeblink responses diverge. These authors also pointed out possible sequential processes that might be related to the orienting reflex.

Barry integrated parts of Sokolov's, Maltzman's, Bernstein's and others' findings about the orienting reflex into the *Preliminary Process Theory* (for a review of its development see [34]). This theory associates responses of the different peripheral measures with different stages of information processing. The first stage of information processing, *stimulus registration*, is modulated by *vigilance* (an “attentive preparatory state”) and is associated with cardiac deceleration (and “cephalic vasodilation”). The next stage comprises *magnitude registration* (associated with peripheral vasoconstriction) and *novelty registration* (associated with respiratory pause). The actual mechanisms of the *orienting reflex*, which depend on novelty, intensity, and significance of the stimulus, present the last stage, which is directly linked to phasic skin conductance responses. The interaction between the partial mechanisms of the orienting reflex is still under discussion [34]. In the Preliminary Process Theory, all stages are framed by the modulatory influence of *cortical sets*. *Cognitive*, *perceptual* and *motor processes* (associated with cardiac acceleration) are the output of this information processing model. The Preliminary Process Theory is mainly based on empirical data from the peripheral nervous system; it describes rather basic processes that are assumed to *precede* cognitive functions.

We argue that these features render the Preliminary Process Theory a suitable framework for understanding why and how physiological responses are an important marker for differentiating true from false memories.

To summarize, a stimulus that a person has already encountered bears higher significance than a completely unknown stimulus. According to the Preliminary Process Theory, this difference in significance should most directly be reflected in differences in phasic electrodermal responses to the presentation of the stimulus. We therefore hypothesize that stimuli that have been falsely recognized bear less significance than comparable correctly recognized stimuli. This difference in significance should be reflected in smaller electrodermal responses for falsely as compared to truly recognized stimuli.

The influence of stimulus significance and novelty on physiological responses were investigated systematically in the context of an information detection paradigm (e.g. [36]–[42]), the Concealed Information Test (CIT; formerly called Guilty Knowledge Test; [43]).

The CIT is a systematic and standardized test procedure comparing the physiological responses of a subject towards a number of crime-relevant yes-or-no questions (e.g. “Have you seen this object?”). Physiological data usually comprise channels such as electrodermal activity, electrocardiogram, breathing activity, and finger plethysmogram. Each crime-relevant question is combined with the presentation of one previously encountered “probe” item and a number of “irrelevant” items. “Irrelevant” items are categorically related to the “probe” item but unknown to the subject. For theory and application of the CIT, see [1], [42].

In a CIT study, responses to known objects (probe items) are compared with responses to unknown objects (irrelevant items). A typical response pattern shows greater electrodermal activity (EDA), lower respiration line length, lower phasic heart rate, and lower finger pulse waveform length [44], [1], [45]–[47], [41] for known objects. This effect is commonly attributed to stimulus significance [1].

In general, CIT studies show that a subject's knowledge about an item influences his/her psychophysiological responses when confronted with it a second time. CIT studies have been successfully conducted with a variety of stimuli, such as words, pictures, cards, and pictures of faces (for a review see [1]).

The idea of examining false memories by means of peripheral physiological measures with application of CIT methodology has already been investigated in a doctoral thesis by Amato-Henderson [48], which used the *misleading information paradigm* [49], [50]. The author reported that misled subjects had a higher probability of being categorized as truthful. Dockree et al. [51] used the misleading information paradigm to elicit false recall in patients with traumatic brain injury and healthy controls. EDA responses were recorded during recall. Results for the healthy control group indicated a relationship between EDA responses and uncertainty during recall. Allen and Mertens [15] combined the DRM paradigm with an event-related potentials-based CIT; they found “little evidence that brain electrical activity could differentiate true from false memories”.

### Aims of the present study

The present study was designed to explore whether responses in peripheral psychophysiology differ between true and false memories. This was implemented by combining a DRM paradigm with electrodermal, cardiovascular, and respiratory measurement. Operationalization and data reduction were performed according to CIT standards. We hypothesized that response differences between false and true recognition would be observable, analogously to the response differences to irrelevant (unknown) vs. probe (known) items in the CIT. Therefore, we expected false memories to elicit lower EDA, higher respiration line length, higher phasic heart rate, and higher finger pulse waveform length than true recognition.

Further aims, as methodological advances over Miller and Gazzaniga's [9] study design, were the introduction of a control group, the introduction of unrelated items, and the use of equal modalities (i.e. pictures only) in study and recognition phase.

We argue that the introduction of a control group is crucial for a false memory study using psychophysiological measures and a “classical” DRM design. Between-group interaction analyses were planned to control for possible *a priori* memorability differences between related lures and studied items. Unrelated items were introduced as a manipulation check of recognition judgments, similarly to previous DRM studies (see [2]). Additionally, it was intended to disentangle electrodermal responses related to orienting and motor activity. For this purpose, a time delay of six seconds was inserted between item presentation and prompt to answer.

## Materials and Methods

### Ethics Statement

This study was approved by the Institutional Review Board of the Institute for Frontier Areas of Psychology and Mental Health (Freiburg, Germany) where the study was conducted. Procedures and measures were explained to the participants. Written informed consent was obtained from all subjects.

### Participants

Sixty students of various faculties voluntarily participated in the study. Exclusion criteria were: insufficient language skills, academic experience in psychology or cognitive science, prior participation in an experiment in the same laboratory. Two subjects had to be excluded from the analyses: one due to alertness problems, one due to mental health problems. The remaining fifty-eight subjects (22 m, 36 f; age 24.3±3.4 years; 52 right-handed) were healthy and unmedicated. All subjects received a compensation of twelve Euros for their participation.

### Groups

Subjects were assigned pseudo-randomly and without the knowledge of the experimenters to either the control group or the experimental group. The final sample consisted of twenty-nine subjects per group. Subjects of the experimental group viewed visual scenes in each of which one particular object, the “related lure”, had been removed; subjects of the control group viewed the complete scenes. The objects omitted in the experimental group will be referred to as “related controls” in the control group, because there they did not serve as “lures”.

### Stimuli

The present experiment used 13 digitalized color paintings from the former American weekly periodical “The Saturday Evening Post” showing stereotypical everyday scenes (e.g. a cleaning scene with a mother and a child displaying amongst other things a shovel, a broom, an apron, and a bin). Even though the present study's learning phase was strongly inspired by Miller and Gazzaniga [9], we only partially used the same scenes (see Appendix S2).

All visual scenes had a resolution of 500*500 pixels. For the control group, all scenes remained untouched, while for the experimental group, related lures were removed. Related lures were chosen in terms of high relatedness to its category. The empty surfaces resulting from this process were digitally retouched. One additional visual scene was prepared analogously for a training study phase.

For the recognition phase, all related lures and studied items were cut out of their respective scene; all unrelated items were extracted from scenes taken from other volumes of “The Saturday Evening Post” (see Appendix S1). Presentation angles and aspect ratios of all extracted items were maintained. Each item was magnified to a maximum size of 300 pixels, put on a white background, and inserted in a white frame with a width of a 100 pixels per side. The resulting pictures, 500*500 pixels in size, were surrounded by a grey presentation mask and presented foveally on a 19″ monitor at a distance of 90 cm. Picture size was 11.95° of visual angle in both dimensions. Each item category related to a visual scene comprised three studied items, two unrelated items and one related lure/related control. A training recognition phase consisting of one unrelated item and two studied items was also prepared.

### Procedure

Similar to several other DRM studies [6], [9], [13], we tried to disguise the study's true nature in order to avoid possible effects of forewarning (see [52]–[54]). As a cover story, we advertised the study as a “series of experiments about social perception and emotion” and introduced an irrelevant valence-rating task in the study phase; in a retention interval, participants filled out a personality questionnaire (included for exploratory purposes, results are not reported here; Tellegen Absorption Scale; [55]; German Version [56]). The reason for the use of a cover story was explained to all subjects after completing the experiment.

1. Study phase: subjects were led to an acoustically and electrically shielded, video surveilled, and dimly lit experimental chamber (*Industrial Acoustics GmbH*, Niederkrüchten, Germany) and seated in front of a monitor; there they received a written instruction for the study phase, asking them to first read the title heading (e.g. “cleaning”) of each picture before taking a thorough look. They were also asked to rate the pictures (see cover story) on a rating scale ranging from 1 “very unpleasant” to 7 “very pleasant” as soon as the rating scale appeared on the screen. After a short training phase, the main run, in which all thirteen visual scenes were presented for 50 seconds in a pseudo-randomized order, was conducted. The rating scale was presented after 40 seconds and remained until confirmed by the participant. A gray screen was presented for four seconds prior to each trial.
2. Retention interval: after a short pause, participants were asked to fill in the Tellegen Absorption Scale, which was announced as the second experiment (see cover story). The mean duration of the retention interval (defined as time between the end of the study phase and the begin of the recognition phase) was 27 min (SD = 4:50 min).
3. Recognition phase: subjects were then led back to the experimental chamber and connected to the polygraph leads. A written instruction asked them to decide, if the following pictures had been included in a visual scene from the “first experiment”. They were instructed to first read the title heading of each picture announcing the different scenes (e.g. “cleaning”). A grey question box saying “Did you see this object?” was presented together with each title heading. Then they had to a look at the presented picture (e.g. a broom) and give a yes-or-no answer by pressing the respective key on the keyboard as soon as two indication fields appeared on the screen. Answers had to be given as quickly as possible by pressing one of the two response keys. Key assignment was balanced across subjects. The given “yes” or “no” answer was marked at the respective indication field and remained visible on the screen as long as the item question was presented.

The main run commenced after a short training phase. The entire presentation time for each item, title, and question box was 11 seconds. The indication fields were presented six seconds after trial onset and stayed until the end of the trial. Each trial was followed by an interstimulus interval of 5±2 seconds (jitter); resulting in a stimulus onset asynchrony of 16 to 18 seconds. The order of the presented categories was identical to the study phase. Related lures/related controls were never presented first; the first item of each category was discarded from evaluation.

**Figure 1:**
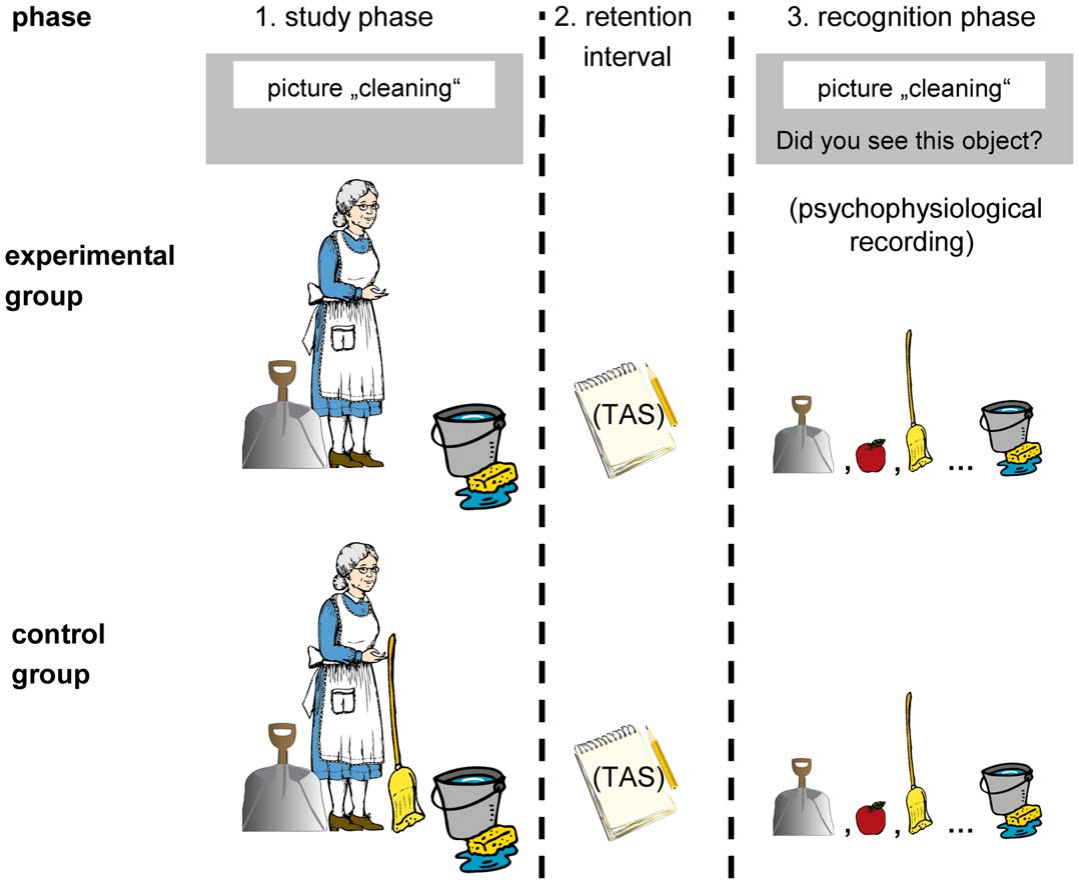
Schematic illustration of all experimental phases. Overview of experimental phases for both groups. In this example, the broom is a related lure (eg)/related control (cg), the shovel and the bucket are studied items, and the apple is an unrelated item. The study phase contained 13 color pictures showing everyday scenes, the recognition phase contained six items per category: three studied items, two unrelated lures and one related lure/related control. Object pictures were downloaded from the “Creative Commons/Public Domain” licensed homepage “www.openclipart.org” and are vicarious for the copyright protected stimuli used in this study (cp. Appendix S1).

The experimental procedure is depicted in figure 1.

### Physiological recording

Subjects sat in an upright position in order to comfortably see the monitor and reach the keyboard. Temperature in the cabin was 21.4±0.9°C at the beginning of the recognition phase's main run, with a maximum increase of 1.6°C over the course of the recording.

Skin conductance, respiratory activity, electrocardiogram, and finger plethysmogram were recorded. Physiological measures were A/D-converted and logged by the *Physiological Data System I 410-BCS* manufactured by *J&J engineering* (Poulsbo, Washington). The A/D-converting resolution was 14 bit, allowing skin conductance to be measured with a resolution of 0.01 µS. All data were sampled with 510 Hz. Triggers indicating question onsets were registered with the same sampling frequency.

For skin-conductance recordings, standard silver/silver chloride electrodes (*Hellige*; diameter 0.8 cm), isotonic signa electrode creme (*Parker Laboratories Inc.*) and a constant voltage of 0.5 volts were used. The electrodes were fixed at thenar and hypothenar sites of the non-dominant hand. For registration of abdominal and thoracic respiratory activity, two PS-2 biofeedback respiration sensor belts (*KarmaMatters*, Berkeley, California) with built-in length-dependent electrical resistances were used. The belts were fixed over clothes at the level of the lower thoracic aperture and the umbilicus, respectively. Electrocardiogram was measured with *Hellige* electrodes (diameter 1.3 cm) according to *Einthoven II*. Finger pulse signal was transmitted by an infrared system in a cuff around the middle finger of the non-dominant hand.

### Behavioral measures

1. Study phase: Behavioral data from the study phase were not analyzed.
2. Recognition phase: After a delay of six seconds between question and prompt to answer, subjects responded with “yes” or “no” by pressing a key. Answers were stored on the stimulus-presenting computer for later evaluation of error rates. The delay was used to prevent confounding orienting-related with motor-related electrodermal responses. Because of the delayed answering, reaction times were discarded from evaluation.

### Data reduction

Electrodermal reactions were assessed with a computerized method based on the decomposition of overlapping reactions as proposed by Lim et al. [57]. The algorithm was adopted from Ambach et al. [44]. The time window used for the definition of the EDA response was defined as 0.5 to 4.5 seconds after item presentation in order to correspond with the first EDA component reported by Ambach et al. [44], which is assumed to reflect orienting-related processes. EDA data from two subjects had to be discarded from analysis, because they met the criterion for hypo-responding defined in this study (more than 90% non-responses).

Respiratory data from both respiration belts were manually scanned and low-pass filtered in order to eliminate artifacts. The total respiration line length was computed over a time interval of 10 seconds after trial onset. The respiration line length measure integrates information about frequency and depth of respiration. The method was derived from Timm [58] and modified by Kircher and Raskin [59]. The respiration line length data from both belts were averaged.

Electrocardiogram data were visually inspected, after notch filtering at 50 Hz and an automatic R-wave peak detection. The R-R intervals were transformed into heart rate and real-time scaled [60]. Heart rate during the last second before trial onset served as pre-stimulus baseline. Phasic heart rate was calculated by subtracting this value from each second-per-second poststimulus value. For extracting the trial-wise information of the phasic heart rate, the mean change in heart rate within 15 seconds after trial onset compared with the prestimulus baseline, was calculated [61], [62].

Finger pulse waveform length within the first 10 seconds after trial onset was calculated from finger pulse waveform and subjected to further analyses [45]. The finger pulse waveform length comprises information about heart rate and pulse amplitude.

### Statistics

Two independent variables determine the design of this study: the within-subject factor “Item Type” (related lures/related controls, studied items, unrelated items) and the between-subjects factor “Group” (experimental group, control group). A third factor, the “Correctness of Response” (true, false) can only be determined item-wise and post-hoc and thus has to be regarded as quasi-experimental. The hierarchical dependency of the data and the unequally balanced cells are major violations of the assumptions of the General Linear Model. Regarding these violations, all calculations of physiological data were made on basis of *Hierarchical Linear Model* analyses. An additional advantage of the Hierarchical Linear Model is that it is able to model individual baseline differences in peripheral physiological data by including random intercepts into the model. This makes within-subject standardization, as proposed by Lykken and Venables [63], dispensable. Therefore, data were not averaged over trials; trials were treated as level 1 units of analysis and subjects as level 2 units of analysis (aggregation variable). Maximum likelihood criteria were employed. “Unstructured” was used as the covariance structure, with 100 iterations being performed. Significance level for the assessment of main and interaction effects was set to 0.05; trends are reported for results below 0.10.

Statistical analyses of recognition judgments include χ^2^ frequency tests to evaluate the frequency distributions across cells. Hereby, different expected values calculated on item presentation frequencies are being considered. A total of nine missing trials (no answer) were removed.

In a first step, direct comparisons of cells were calculated within the experimental group. Then, in order to consider possible systematic differences between item types, analyses of Group by Item Type interaction effects were conducted; Item Type was restricted to the levels studied items and related lures/related controls only. Unrelated items were only used as a manipulation check to test for a possible bias in recognition; physiological data from these items were not analyzed. All statistical analyses were performed with PASW, Version 18.0.0 (*SPSS Inc.*, Chicago).

## Results

### Recognition judgments

**Figure 2:**
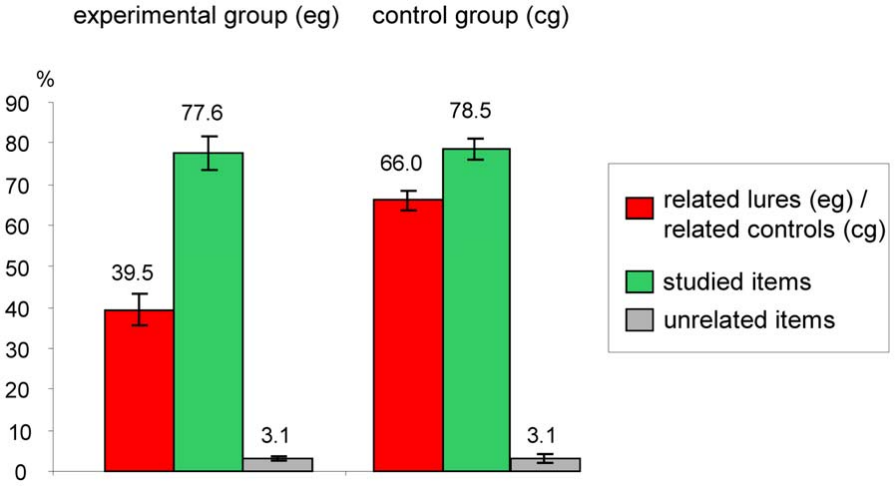
Response behavior in the recognition phase across all item types and groups. Proportion of trials with a “yes” answer. Error bars represent standard errors of the mean.

Figure 2 summarizes response behavior in the recognition phase for both groups. In the experimental group, the proportion of falsely recognized related lures was higher than the proportion of falsely recognized unrelated items (χ^2^ [1, N = 587] = 114.41; p<0.001). In the control group, proportions of recognized related controls and studied items also differed significantly (χ^2^ [1, N = 1254] = 21.91; p<0.001).

### Skin conductance

Hierarchical Linear Model analysis for EDA showed a trend towards smaller response amplitudes for falsely recognized related lures compared to correctly recognized studied items (F [1,714.44] = 2.86; p = 0.09) in the experimental group. The corresponding analysis in the control group showed no effect (F [1,910.30] = 1.01; n.s.).

The 2×2 Hierarchical Linear Model analysis for EDA showed a significant Group by Item Type interaction (F [1,1625.81] = 4.01; p = 0.045), confirming the observed differences between true and false recognition on between-group level.

### Respiration

Respiration line length data showed lower values for falsely recognized related lures than recognized studied items in the experimental group (F [1,802.55] = 4.94; p = 0.027). In the control group, reactions differed analogously between recognized related controls and recognized studied items (F [1,909.16] = 5.085; p = 0.024). No Group by Item Type interaction was found (F [1,1711.72] = 0.00; n.s.), indicating that a difference between true and false recognition was not proven at the between-groups level.

### Heart rate

Regarding phasic heart rate, only a trend was found. In the experimental group, falsely recognized related lures were accompanied by lower phasic heart rate values than correctly recognized studied items (F [1,828.17] = 3.25; p = 0.072). No such effect was found in the control group (F [1,927.54] = 2.52; n.s.), no Group by Item Type interaction (F [1,1760.35] = 0.00; n.s.) was found.

### Finger pulse

For finger pulse waveform length, neither a main effect for the comparison of falsely recognized related lures and correctly recognized studied items in the experimental group, (F [1,782.28] = 0.17; n.s.) nor for the corresponding control analyses in the control group (F [1,909.30] = 0.32; n.s.), nor a Group by Item Type interaction (F [1,1691.60] = 0.20; n.s.) was found.

**Figure 3:**
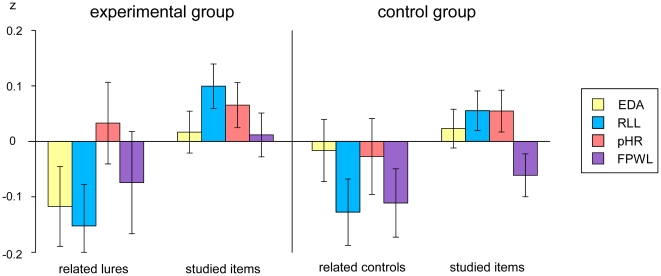
Physiological responses to false and true recognition (z-scores). Overview of electrodermal activity (EDA), respiration line length (RLL), phasic heart rate (pHR) and finger pulse waveform length (FPWL) responses to false and true recognition and their respective control conditions (control group). Error bars represent standard errors of the mean. The physiological measures were z-transformed (for illustration purposes only) for each subject and for each data channel according to [72], [47], [73]. All trials except the first trials of each stimulus category were used for the calculation of individual means and standard deviations.

Figure 3 gives an overview of responses across subjects for all physiological measures.

## Discussion

The main goal of the present study was to examine false recognition with measures of peripheral physiology. Additionally, it aimed at investigating the applicability of scenic pictures as DRM study phase stimuli, with the recognition phase having the same modality as the study phase (cp. [9]).

### Behavioral measures

Response behavior in the recognition task was, with a false recognition rate of 39.5%, smaller than in a previous DRM study with visual scenes ([9]; false recognition rate in the pictorial condition: 50%), but still substantial. As expected, the proportion of false recognition of related lures was distinctly higher than the proportion of falsely recognized unrelated items. This result pattern is typical for DRM studies and points out that combining visual scenes in the study phase with pictorial stimuli in the recognition phase yields behavioral results comparable to previous DRM studies.

Differences between the true recognition rates for related controls and studied items in the control group point to systematic a priori differences of these stimulus types. These are a consequence of the use of a “classical” DRM design because there, related lures are chosen in terms of their forward and backward associative strength [64] and not in terms of being comparable to studied items. The present study featured between-group analyses of psychophysiological measures to control for this bias statistically. Regrettably, no comparisons with previous studies are possible, because “classical” DRM design studies have not reported control group data. Additionally, the direction of this bias points to lower memorability of lures. Knowing this direction is important, since it indicates that the false memory rate in the experimental group is robust and not due to this bias. On the contrary, the false memory rate would have supposedly been higher without this bias.

### Physiological measures

Psychophysiological results showed a significant between-group interaction for EDA. Though respiration line length and phasic heart rate tendentially differed between false and true recognition within the experimental group, the findings for these measures need to be interpreted with care, since only between-group interactions address a-priori stimulus differences. No effects were found for finger pulse waveform length. The reason for this remains unclear; a visual inspection suggests that it might be due to insufficient finger pulse waveform length signal quality in a considerable number of participants.

The lack of significant between-group findings for respiration line length, phasic heart rate, and finger pulse waveform length might have several reasons: Firstly, it might be due to the relatively small number of falsely recognized related lures. In CIT studies, the number of items to be included in the analysis is foreseeable and does not differ greatly between subjects. Yet, this is not the case in DRM studies. Thus, in DRM studies, data representing physiological within-subject differences of some participants tend to be quite noisy. Regarding the CIT, meta-analytic evidence shows that the number of questions (and thus, the number of “probe” items) is positively related to effect size [1]. Secondly, the Preliminary Process Theory predicts that EDA should be most closely related to orienting sub-processes sensitive to stimulus significance. Furthermore, in CIT studies, EDA is consistently the marker with the largest effect sizes [1].

In sum, the relatively small number of falsely recognized related lures might have caused a generally small physiological effect, which was suprathreshold in EDA only, because EDA is most closely related to significance and the strongest marker in CIT studies. In this sense, the study might have been underpowered with respect to cardiovascular and respiratory measures.

The analysis of skin conductance responses showed greater response amplitudes associated with true than false recognition. This can be viewed in the light of the observation from CIT studies that familiar objects evoke stronger orienting reactions and therefore greater skin conductance responses than unknown objects.

The present EDA result could be interpreted according to the Preliminary Process Theory. The Preliminary Process Theory [34] states that orienting is modulated by significance, which can be a product of classical conditioning (through cortical sets). The study phase was presumably associated with additional focused cortical activity (cortical sets; e.g. because of the instruction or schematic knowledge). The studied items (being paired with the cortical sets) acquired significance through classical conditioning. In the recognition phase, the studied items (now conditioned stimuli) elicited significance as conditioned response, which in turn modulated the orienting reflex. The heightened orienting response was thus reflected in greater EDA responses.

The finding of the present study could cautiously be interpreted in that CIT-like differentiations of known and unknown objects are possible, even if a subject is unaware of actually not knowing some of the objects. Possibly, differentiation of true and false recognition by means of skin conductance might be more attributable to the objective knowledge of an object and less to the subjective belief of knowing it. Early EDA studies [65], [66] coining the phrase “subception” have brought forward evidence that implicit knowledge is accompanied by greater EDA responses than lacking knowledge. This is in line with the physiological discrimination of known vs. unknown faces in prosopagnosic patients (e.g. [67]–[69]). For a discussion of the applicability of the CIT in the clinical context and conceptual considerations about implicit knowledge, explicit knowledge and prosopagnosia, see [70].

### Conclusions

The present study introduced two innovations. Firstly, it combined a modified DRM paradigm with recordings of several measures of peripheral physiology. This was mainly carried out by borrowing methodological and theoretical concepts from the CIT paradigm. Still, it is unclear, which CIT-processes can be transferred to false memory research. In the detection of concealed information, the orienting reflex and its modulators *significance* and *novelty* [29] are seen as primarily responsible for the effects reported in CIT studies [40].

It is plausible to assume that true recognition of studied items in DRM studies and true recognition of probe items in CIT studies are both associated with heightened significance. Gati and Ben-Shakhar [38] attempted to clarify the roles of significance and novelty in the orienting reflex by a series of CIT experiments. They stated that “responsivity is positively related to the degree of match between the input and the representation of significance”. A speculative explanation for false recognition being accompanied by smaller EDA responses than true recognition could be that false recognition is accompanied by a higher mismatch between input and the representation.

In other words, a subject gains (mental) representations of the related lures during the study phase. This process is supposedly influenced by schematic knowledge. The representations match poorly to the actual stimuli shown in the recognition phase, while the representations of studied items match relatively well to the (same) stimuli shown in the recognition phase. If this is the case, the lack of significance of falsely recognized related lures would be a suitable explanation for our result.

As a second innovation, this study has been conducted with a study phase with visual scenes and a pictorial recognition phase, ensuring comparable modalities for the encoding and the retrieval phase. We argue that this is an important feature of a visual DRM study, since modalities should be equal to speak of recognition in the literal sense. Equality of modalities might be interpreted as one of Roediger and McDermott's [2] crucial changes of Deese's [71] paradigm.

To conclude, the present study can be regarded as a first step to investigate false memories, as provoked in a visual DRM study, with measures of peripheral psychophysiology. Differences in memorability of stimulus types are an inherent problem of the classical DRM design. In the present study, this problem was brought to light and faced by the introduction of a control group, whereas previous DRM studies did not feature control groups and could therefore not report such a memorability bias.

Future DRM studies with CIT methodology might account for this problem by using a categorical design with a random choice of the related lures. The realization of such a design seems to be challenging with visual scenes as study material, but it might be fruitful.

The present peripheral psychophysiology study combines two fields of research. The phenomena of false memories and deception overlap conceptually. This is particularly important when object recognition is investigated psychophysiologically. The main difference between the two phenomena, false recognition and information concealment, is the level of awareness concerning the falseness of a recognition statement. While the detection of deception has been extensively studied by means of peripheral psychophysiology, this has scarcely been the case for false memories.

However, the present study not only contributes to the understanding of false memories, but from a detection-of-information perspective, the results also provide evidence for the detectability of implicit knowledge. Thus, it might also be seen as an applied investigation of “subception”. *Implicit* knowledge might contribute greatly to the psychophysiological detectability of deception; there, implicit and explicit knowledge are confounded.

The detectability of implicit knowledge has important practical implications. The question whether a subject carries (detectable) information about an encounter, without having conscious access to it, arises in different fields of interest. Regarding autobiographical memories, future applications of psychophysiological methods might help to distinguish true from false “recovered memories”. A future development of psychophysiological methods for the detection of implicit knowledge might also be interesting for medical rehabilitation, e.g. regarding amnesia. We must however emphasize that these implications are rather speculative.

To summarize, our results show that true recognition is accompanied by higher EDA responses than false recognition.

## Supporting Information

Appendix S1 **Stimulus material for the study phase (visual scenes) and extracted related lures/related controls and studied items (recognition phase).**
(DOC)

Appendix S2 **Additional stimulus material for the recognition phase (unrelated lures).**
(DOC)
