# Supplementary material for: Psychophysiology of False Memories in a Deese-Roediger-McDermott Paradigm with Visual Scenes
Source: PLoS One. 2012 Jan 17;7(1):e30416. doi: 10.1371/journal.pone.0030416 (PMC3260301; doi:10.1371/journal.pone.0030416)
Supplement: Appendix S1 — Stimulus material for the study phase (visual scenes) and extracted related lures/related controls and studied items (recognition phase). (DOC) [file pone.0030416.s001.doc]

| category no. | working title for visual scene | volume  (The Saturday Evening Post) | page | related lures  / related controls | studied items | | |
| --- | --- | --- | --- | --- | --- | --- | --- |
| 1 | fish vendor | 7/2/1949, 222(1) | cover | hand net | scale | rubber boots | crab |
| 2 | cleaning | 4/2/1955, 227(40) | cover | shovel | bin | broom | pinafore |
| 3 | baking | 5/21/1955, 227(47) | cover | oven | food processor | cake | frosting bowl |
| 4 | artist | 6/6/1953, 225(49) | cover | palette | easel | artist case | brush |
| 5 | music session | 12/1/1956, 229(22) | cover | cymbal | trumpet | trombone | drum |
| 6 | tent camp | 8/1/1953, 226(5) | cover | paddle | canoe | totem pole | tent |
| 7 | plumber | 6/2/1951, 223(49) | cover | plunger | pipe wrench | toolbox | suction cup |
| 8 | blacksmith | 11/2/1940, 213(18) | 11 | horse shoe | hammer | anvil | apron |
| 9 | restaurant | 11/6/1954, 227(19) | cover | coffee cup | bow tie | bread basket | salad plate |
| 10 | paperhanging | 11/26/1949, 222(22) | cover | wallpaper brush | scissors | bucket | ladder |
| 11 | tooth brushing | 1/29/1955, 227(31) | cover | tooth mug | pajama pants | towel | mirror |
| 12 | golf course | 8/31/1957, 230(9) | cover | golf caddy | golf irons | gulf cap | flagstick |
| 13 | school class | 2/25/1956, 228(35) | cover | inkpot | pen | notepad | book |
|  |  |  |  |  |  |  |  |
| training phase | barber | 2/27/1943, 215(35) | cover | - | shaver | glasses | - |
