# Supplementary material for: Psychophysiology of False Memories in a Deese-Roediger-McDermott Paradigm with Visual Scenes
Source: PLoS One. 2012 Jan 17;7(1):e30416. doi: 10.1371/journal.pone.0030416 (PMC3260301; doi:10.1371/journal.pone.0030416)
Supplement: Appendix S2 — Additional stimulus material for the recognition phase (unrelated lures). (DOC) [file pone.0030416.s002.doc]

| used in category no. | unrelated lure | disamb-iguation | volume  (The Saturday Evening Post) | page |
| --- | --- | --- | --- | --- |
| 1 | reading-glass | left | 2/27/1954, 226(35) | cover |
| 1 | balloon | - | 11/2/1957, 230(18) | cover |
| 2 | aquarium | - | 4/4/1959, 231(40) | cover |
| 2 | bird house | - | 3/19/1955, 227(38) | cover |
| 3 | handbag | - | 3/21/1959, 231(38) | cover |
| 3 | silhouette | - | 11/21/1953, 226(21) | cover |
| 4 | lemonade | top | 11/19/1960, 233(21) | cover |
| 4 | watering can | left | 6/4/1955, 227(49) | cover |
| 5 | bike | right top | 8/14/1954, 227(7) | cover |
| 5 | vacuum flask | - | 5/30/1953, 225(48) | cover |
| 6 | package | - | 12/17/1960, 233(25) | cover |
| 6 | Lawn mower | - | 5/14/1955, 227(46) | cover |
| 7 | cheese rasp | - | 12/5/1953, 226(23) | cover |
| 7 | gloves | - | 1/4/1958, 230 (27) | cover |
| 8 | alarm clock | - | 7/23/1938, 211(4) | cover |
| 8 | sock | left | 12/11/1954, 227(24) | cover |
| 9 | Christmas tree | - | 12/25/1954, 227 (26) | cover |
| 9 | peaked cap | - | 12/18/1937, 210(25) | cover |
| 10 | hat | - | 12/17/1960, 233(25) | cover |
| 10 | bedside lamp | - | 7/23/1938, 211(4) | cover |
| 11 | baseball cap | - | 5/1/1954, 226(44) | cover |
| 11 | rolling pin | - | 2/26/1955, 227(35) | cover |
| 12 | parasol | (middle; rotated: 90°, clockwise) | 7/10/1954, 227(2) | cover  cover |
|
| 12 | bus | - | 9/17/1955, 228(12) | cover |
| 13 | tennis racket | - | 8/6/1938, 211( 6) | cover |
| 13 | lantern | - | 9/25/1954, 227(13) | cover |
|  |  |  |  | cover |
| training phase | hanging banner | - | 1/26/1946, 218(30) | cover |
